# Spatial distribution, prevalence and diversity of haemosporidians in the rufous-collared sparrow, *Zonotrichia capensis*

**DOI:** 10.1186/s13071-018-3243-4

**Published:** 2019-01-03

**Authors:** Daniela Doussang, Daniel González-Acuña, Luis Gonzalo Torres-Fuentes, Stephen C. Lougheed, Rute Beatriz Clemente-Carvalho, Kian Connelly Greene, Juliana A. Vianna

**Affiliations:** 1 Facultad de Ciencias Veterinarias, Universidad de Concepción, Casilla, 537 Chillán, Chile; 2 Departamento de Ecosistemas y Medio Ambiente, Facultad de Agronomía e Ingeniería Forestal, Pontificia Universidad Católica de Chile, Código Postal: 6904411, Casilla 306, Correo, 22 Santiago, Chile; 3 Department of Biology, Queen’s University, Kingston, ON K7L 3N6 Canada

**Keywords:** Avian malaria, Avian host, *Plasmodium*, *Haemoproteus*, Altitude, Latitude

## Abstract

**Background:**

Parasite prevalence and diversity are determined by the distribution of hosts and vectors and by the interplay among a suite of environmental factors. Distributions of parasite lineages vary based on host susceptibility and geographical barriers. Hemoparasites of the genera *Haemoproteus* and *Plasmodium* have wide distributions, and high prevalence and genetic diversity within perching birds (Order Passeriformes). The rufous-collared sparrow (*Zonotrichia capensis*) is widely distributed in Central and South America across an immense diversity of environments from sea level to more than 4000 meters above sea level. It therefore provides an excellent model to investigate whether altitudinal and latitudinal gradients influence the distribution, prevalence and diversity of haemosporidian parasites, their population structure and the biogeographical boundaries of distinct parasite lineages.

**Results:**

We assembled samples from 1317 rufous-collared sparrows spanning 75 locales from across Central and South America (between 9.5°N and 54°S; 10–4655 meters above sea level). We used DNA sequence data from a fragment of the mitochondrial cytochrome *b* gene (*cytb*) of *Haemoproteus* and *Plasmodium* from 325 positive samples and found prevalences of 22 and 3%, respectively. *Haemoproteus* exhibited a higher prevalence than *Plasmodium* but with comparatively lower genetic diversity. We detected a relationship of *Plasmodium* and *Haemoproteus* prevalence with altitude and latitude; however, altitude and latitude did not influence parasite diversity.

**Conclusions:**

Parasite lineages showed a phylogeographical boundary coincident with the Andes Mountains, although we also observed a north-south disjunction in Peru for *Haemoproteus*. Haemosporidian distribution was not homogeneous but differed based on latitude and altitude. This is most probably due to environmental factors that have influenced both vector distribution and abundance, as well as parasite development. Our study provides key insights on the distribution of haemoparasite lineages and parasite dynamics within hosts.

**Electronic supplementary material:**

The online version of this article (10.1186/s13071-018-3243-4) contains supplementary material, which is available to authorized users.

## Background

In a rapidly changing world with many newly-emerging or geographically-expanding pathogens and parasites, we must investigate factors implicated in distribution of these organisms. Avian haemosporidia (*Plasmodium*, *Haemoproteus*, *Leucocytozoon* and *Fallisia*) are a group of blood parasites transmitted by vectors [1] and, due to their complex life-cycles, the prevalence, diversity, and distribution of these taxa are influenced by a dynamic interplay among hosts and their environment [2, 3]. Ecological factors such as the distribution, abundance and species richness of intermediate (birds) and definitive hosts (Diptera) regulate the transmission possibilities of hemoparasites [4, 5] and can promote their diversification. These ecological factors, in turn, may be influenced by the geography and evolutionary history of the hosts, providing opportunities to understand how host-parasite interactions influence parasite diversity [6, 7].

The distribution of avian haemosporidians differs among zoogeographical regions (Holarctic, Ethiopian, Oriental, Australian, Neotropical and Antarctic) [1]. The level of phylogeographical structure depends on the factors that most strongly influence parasite distributions and, in particular, we predict that such structure will be present if distributions are more related to factors like vector diversity and habitat heterogeneity [6]. Biogeographical patterns for distribution, prevalence and diversity of haemosporidian parasites have been described for multiple regions worldwide. Prevalences for both genera (*Haemoproteus* and *Plasmodium*) have been shown to be lower at higher altitudes [8, 9], with a greater limitation of *Plasmodium* at higher altitudes [10, 11].

Climate is closely linked to altitude and latitude, with lower temperatures occurring at higher altitudes and latitudes that could result in slower developmental rates of both parasite and vector [1]. Therefore, vector-borne diseases could impact hosts differently at different elevations, as rates of vector development and distribution could either limit or facilitate parasite transmission [10]. Thus, we expect that latitude may also relate to the presence of avian haemosporidians [12]. For example, the prevalence and diversity of these parasite lineages has been shown to increase at lower tropical latitudes [13–15]. In contrast, in a meta-analysis, Clark [16] found no correlation between parasite diversity and latitude worldwide; however, this study did not include considerations of avian host species in their analyses. This is a crucial factor since haemosporidia lineage diversity should relate to the density of susceptible avian hosts and to parasite-host specificity [17, 18]. Globally, *Haemoproteus* exhibits greater lineage diversity than *Plasmodium*; however, this pattern differs in South America, where a higher avian host diversity coupled with low *Plasmodium*-host specificity leads to greater lineage diversity of *Plasmodium* than *Haemoproteus* [15]. *Haemoproteus* lineages exhibit greater host specificity than *Plasmodium* lineages due to their high vector specialization on ceratopogonid and hippoboscid flies [1]. Several lineages of *Plasmodium* show extreme generalist host-parasitism strategies, while others appear to be restricted to particular host families over recent evolutionary history [4].

The rufous-collared sparrow is one of the most broadly-distributed passerines in the world, with a geographical range that spans the Americas from southern Mexico to Cape Horn (southern Chile) [19]. In the Southern Cone, they occur in an impressive diversity of environments, including coastal habitats, lowland desert, Patagonian steppe, scrub, grassland, Andean desert, forest, valley, and thorn scrub [20, 21]. This broad geographical range and habitat diversity makes this species an excellent subject for evaluating how habitat, latitude, altitude and evolutionary history might shape parasite prevalence and diversity. The evolutionary history of rufous-collared sparrows was influenced by major Pleistocene biogeographical events resulting in three main haplogroups: (i) spanning Central America, the Dominican Republic and north-western South America; (ii) encompassing the Dominican Republic, Roraima (Venezuela), La Paz (Bolivia) and south of Tierra del Fuego, Argentina; and (iii) eastern Argentina and Brazil [22]. Rufous-collared sparrows exhibit a great diversity of *Haemoproteus* and *Plasmodium* in Chile and other areas of South America [9, 13, 23–30].

Previous studies of avian haemosporidians in wild birds have evaluated the phylogeny of the parasites, and tested for the possible effects of altitude [3, 10, 24] and latitude [16, 23] on haemosporidian diversity and prevalence. These studies, however, typically focused on small study areas and multiple avian host species, precluding evaluation of how environmental and evolutionary factors shape patterns within one avian host. In the present study, we investigate the distribution and prevalence of haemosporidians in a broad area of study and in a species-specific host. We hypothesized that haemosporidian distributions are shaped by both the evolutionary history of the avian host and the recognized biogeographical barriers in Central and South America. Furthermore, we hypothesized that haemosporidian distributions would show different prevalence and diversity across latitudinal and altitudinal environmental gradients. We predicted that parasite prevalence would vary with latitude for both genera and that *Plasmodium* would be restricted to lower altitudes relative to *Haemoproteus*. Differences in prevalence and genetic diversity of *Haemoproteus* and *Plasmodium* associated with altitude and latitude would also imply adaptation of these parasites to local environmental conditions.

## Methods

### Study area

**Fig. 1:**
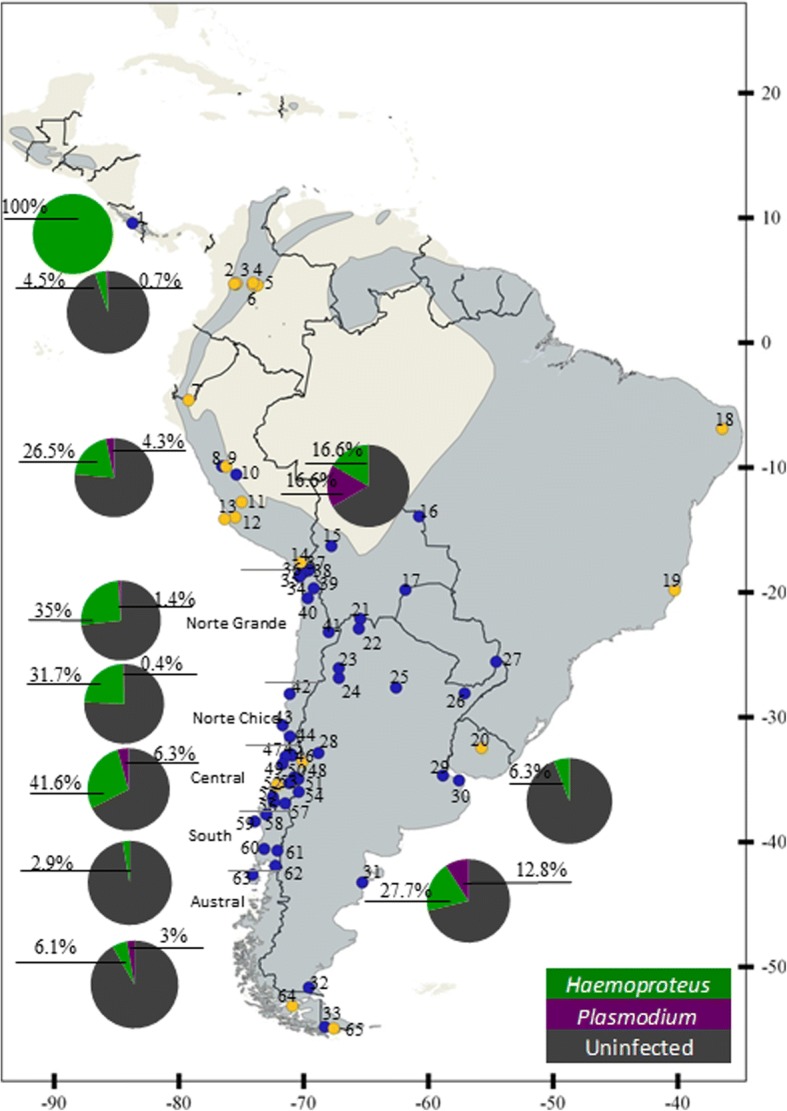
Map of sampling localities and prevalence according to sampling area. Map of South America indicating sampled geographical locations (blue dots) and of other studies (yellow dots); numbers close to dots represent the number of the sample site (Additional file 1: Table S1). Distribution of rufous collared sparrows (*Zonotrichia capensis*) (light gray) is based on BirdLife International data. Pie charts exhibit prevalence of *Haemoproteus* (green) and *Plasmodium* (purple) and uninfected (gray) by sampling area

We used a total 1317 samples of rufous-collared sparrow from 75 locations in Central and South America. Blood samples of 531 rufous-collared sparrows were collected during the period 2010–2016 from 29 localities across Chile, and these were combined with 59 other samples from 19 localities in Costa Rica, Bolivia, Peru and Argentina. An additional 727 samples from other locations that had already been assessed for haemosporidians were added from previous studies (Fig. 1, Additional file 1: Table S1 and Additional file 2: Table S2). Our 1317 samples thus span an extensive latitudinal (9.5°N to 54°S) and altitudinal (10–4655 meters above sea level, masl) range, which we used to quantify diversity and determine phylogeographical patterns and boundaries.

### Sample collection

Adult birds were captured using mist nets in Chile. Blood samples were collected by puncturing the brachial vein [31] and 30–50 μl of blood was obtained and preserved in 1.5 ml microcentrifuge (Ependorff tube) tubes with 96% ethanol until subsequent processing in the laboratory. The rufous-collared sparrow samples from other countries (Costa Rica, Bolivia, Peru and Argentina) comprised 46 blood samples and 13 muscle, liver or heart tissues from specimens that were prepared as study skins for museum collections (Louisiana State Museum, USA).

### DNA extraction, PCR amplification and sequencing

DNA was isolated using a salt extraction method developed by Aljanabi & Martínez [32]. DNA quality and concentration (ng/μl) were estimated using a NanoDrop 2000c spectrophotometer (Thermo Scientific, Waltham, Massachusetts, USA). We amplified a 533 bp fragment of the mitochondrial cytochrome *b* gene (*cytb*) of focal *Haemoproteus*/*Plasmodium* parasites using non-specific primers 3760F (5'-GAG TGG ATG GTG TTT TAG AT-3') and 4292Rw (5'-TGG AAC AAT ATG TAR AGG AGT-3') [33].

Polymerase chain reaction (PCR) reactions were carried out in final volumes of 30 μl, comprising 2 μl of template DNA, 1× reaction buffer, 1.5 mM MgCl_2_, 0.2 mM of each dNTP, 0.5 μM of each primer, and 1.25 units of Taq Platinum (Invitrogen, Carlsbad, California, USA). All PCR reaction sets included negative (ddH_2_O) and positive controls (samples previously confirmed by sequencing and microscopy). The PCR amplification profile was as follows: initial denaturation at 94 °C for 2 min; 40 cycles of denaturation at 95 °C for 40 s, annealing at 52 °C for 1 min and extension at 72 °C for 1 min; with a final extension at 72 °C for 10 min. PCR products were visualized using electrophoresis on 0.8% agarose gel with SB 1× buffer with GelRed™ [34]. Three different PCR reactions were conducted for each sample: one with isolated DNA template without controlling for concentration, and two other reactions with DNA concentrations of 50 and 20 ng/μl, respectively. Samples were considered positive when the parasite DNA was amplified in one of these three reaction conditions.

PCR products were purified and sequenced by Macrogen (Seoul, Korea). Sequences were edited and aligned using Sequencher v.5.4.5 (Gene Codes Corporation, Ann Arbor, Michigan, USA), and polymorphic sites were identified with ClustalX2.1 [35]. Haplotypes were identified using DNAsp v.5.10.1 software [36].

### Prevalence, genetic diversity and population genetic structure

For prevalence estimates, five samples were excluded due to the lack of appropriate positive and negative controls in a previous study, leaving a total of 1312 samples. The prevalence of haemosporidian infection was calculated for all bird samples combined, as well as for each geographical region. Parasite prevalence for each sampling area was estimated as: *P* = number of infected hosts/number of sampled hosts × 100, using Excel software. The pooled prevalence for generalized linear model analyses was determined with 95% confidence intervals (CI) using the function binom.test (number of infected hosts, number sampled hosts, 0.5, alternative="two.sided", conf.level = 0.95) in R studio 386 3.0.1 [37].

Sampling locations were grouped according to country, with Chile being further subdivided into five natural geographical zones (Norte Grande, Norte Chico, Central, South and Austral) which corresponded to a north-south gradient of humidity varying from the Atacama Desert, through Mediterranean ecosystems, to temperate rainforest (see Table 1, Additional file 1: Table S1).

**Table 1:** Genetic diversity from cytochrome *b* sequences of *Haemoproteus* and *Plasmodium* by locality from 325 samples

Location	*N*	*Haemoproteus*					*Plasmodium*				
		*N*+	nH	S	Hd	π	*N*+	nH	S	Hd	π
Costa Rica	2	2	2	17	1	0.039	0	–	–	–	–
Colombia	428	19	3	9	0.578	0.003	3	3	2	1	0.003
Ecuador	1	1	1	0	1	0	0	–	–	–	–
Peru	211	56	3	11	0.284	0.006	9	4	36	0.694	0.039
Bolivia	6	1	1	0	1	0	1	1	0	1	0
Brazil	4	–	–	–	–	–	4	3	17	0.833	0.019
Uruguay	16	–	–	–	–	–	1	1	0	1	0
Argentina	47	13	2	10	0.282	0.006	6	4	46	0.866	0.049
Norte Grande Chile	140	49	4	33	0.157	0.006	2	2	1	1	0.002
Norte Chico Chile	187	48	1	0	0	0	1	1	0	1	0
Central Chile	207	94	2	17	0.082	0.003	11	3	33	0.618	0.039
South Chile	35	1	1	0	1	0	0	–	–	–	–
Austral Chile	33	2	2	12	1	0.027	1	1	0	1	0
Total	1317	286	10	50	0.325	0.008	39	18	68	0.931	0.050

*Abbreviations:* N, total number of samples; N+, number of positive samples; nH, number of haplotypes found; S, number of polymorphic sites; Hd, haplotype diversity; π, nucleotide diversity

Genetic diversity was measured for each geographical region using number of polymorphic sites (S), haplotype number (h), gene diversity (Hd), and nucleotide diversity (π) of *cytb* for both *Haemoproteus* and *Plasmodium* in Arlequin v.3.5 software [38]. Pairwise F_ST_ and Φ_ST_ were calculated between all location pairs to test for the signature of population differentiation. We also performed a Bayesian analysis of the population structure for *cytb* sequences using Bayesian Analysis of Population structure v.6 (BAPS) (http://www.helsinki.fi/bsg/software/BAPS/). This program partitions individuals into groups using maximum likelihood [39]. We used spatial cluster of group, ordering the lineages with the geographical coordinates of the localities where they were detected.

### Biogeography and parasite distribution

We applied generalized linear models (GLMs) to identify possible effects of the latitude and altitude (explanatory variables) on the prevalence of infection and lineage genetic diversity such as haplotype and nucleotide (response variables). We evaluated each genus separately (*Haemoproteus* and *Plasmodium*) in R studio 386 3.0.1 [37] using GLM with a binomial error structure for prevalence and Poisson error for genetic diversity. All GLMs were subjected to residual analyses to evaluate the adequacy of the error distribution. For prevalence we included data from all locales with the exception of Costa Rica, Brazil, Bolivia and Ecuador because of the small sample sizes. Samples were grouped by country (according to geographical areas of sampling), and for Chile the aforementioned geographical areas were separated following a latitudinal gradient.

### Phylogenetic analysis

The parasite sequences for our study were compared to other South America mtDNA *cytb* sequences using data available in MalAvi [14] and GenBank. The best nucleotide substitution model (GTR + I + G) was determined using JModeltest v.2.1.3 [40], applying both AIC (Akaike information criterion) and BIC (Bayesian information criterion) for *Haemoproteus* and *Plasmodium* separately.

To evaluate the relationship between the parasite haplotypes and clades with the geographical distribution and the Andes as a geographical boundary, we performed phylogenetic reconstruction in MrBayes v.3.1.2 [41]. We used 28 sequences (441 bp) in addition to *Leucocytozoon toddi* as an outgroup. The analysis was run for one million generations, sampling every 1000 generations to create a consensus tree; the standard deviation of the split criterion was less than 0.01. We considered nodes with posterior probabilities of 90% or more on the consensus tree to be robust support. The phylogeny was visualized using FigTree v.1.3.1 [42]. To further visualize the relationships among haplotypes and to evaluate genetic distinctiveness, we created a median-joining network using Network v.5.0 [43].

## Results

### Parasite prevalence, diversity and distribution

We found 325 rufous-collared sparrows that were positive for haemosporidian infection out of the total 1317, spanning 75 studied localities. This corresponded to 25% of all cases of *Haemoproteus* (*n* = 286) and *Plasmodium* (*n* = 39) detection. Prevalence differed markedly between genera.

Considering all of the data, the lowest prevalence was evident in Colombia for both *Haemoproteus* and *Plasmodium*, while in Peru, Argentina and Chile a higher prevalence was detected for *Haemoproteus* relative to *Plasmodium.* In Costa Rica and Bolivia, prevalence of *Haemoproteus* was high but sample sizes were small. In Costa Rica and Uruguay we found no *Plasmodium*. *Haemoproteus* showed low prevalence in Uruguay (6.3%) (Fig. 1). In Chile, we observed the highest prevalence of *Haemoproteus* in central (42%) and northern Chile (35%), with a low prevalence in the southern (2.8%) and austral (6%) areas (Additional file 1: Table S1). For *Plasmodium*, the highest prevalence occurred in Argentina (12.8%), central Chile (6.3%) and Peru (4.3%) (Fig. 1).

**Fig. 2:**
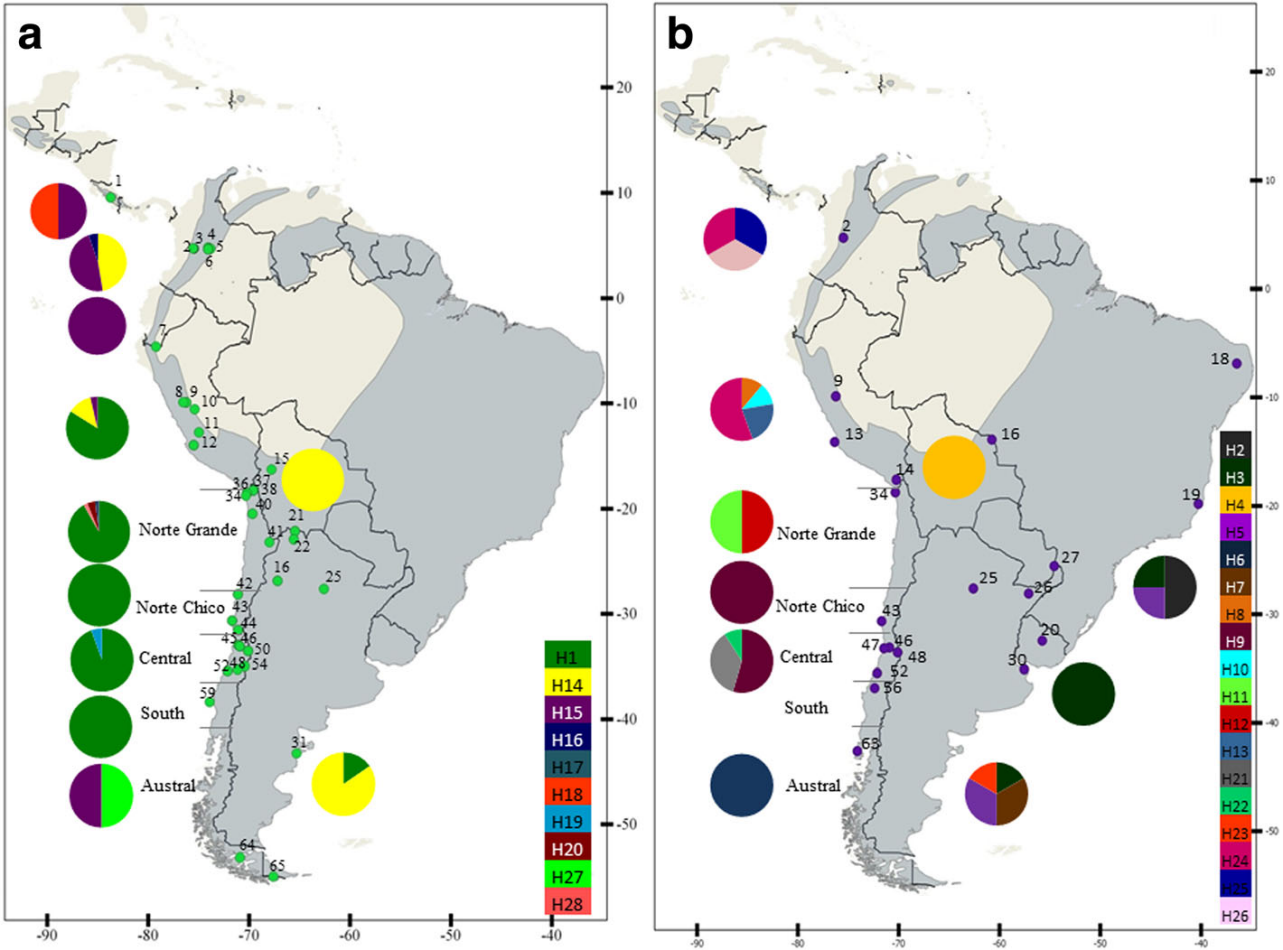
Map of sampling localities and diversity for *Haemoproteus* (**a**) and *Plasmodium* (**b**). Map of South America indicating geographical locations with *Haemoproteus* positive samples (green dots) and *Plasmodium* positive samples (purple dots) (Additional file 1: Table S1); distribution of rufous collared sparrows (*Zonotrichia capensis*) (light gray) is based on BirdLife International data. Pie charts exhibit diversity of *Haemoproteus* (**a**) and *Plasmodium* (**b**)

We identified a total of 28 parasite lineages based on 441 bp of *cytb*: 10 lineages of *Haemoproteus* and 18 lineages of *Plasmodium*. One *Haemoproteus* haplotype (haplotype 1) was the most frequent throughout the entire distribution; it was found in 233 of 325 positive samples (Fig. 2, Additional file 2: Table S2). This haplotype was found to be distributed from Peru, throughout all of Chile (except the austral location) and Argentina. All other *Haemoproteus* and *Plasmodium* lineages were found in only one or two rufous-collared sparrow individuals. The highest number of *Haemoproteus* haplotypes was found in Socoroma, in the north of Chile (18°S).

*Plasmodium* showed a comparatively higher haplotype and nucleotide diversity (Hd = 0.931, π = 0.050) than *Haemoproteus* (Hd = 0.325, π = 0.008). *Haemoproteus* exhibited greater haplotype diversity at lower latitudes, decreasing toward southern Colombia (0.578), Peru (0.284), Argentina (0.282), Norte Grande, Chile (0.157), Norte Chico (0) and central Chile (0.082). The nucleotide diversity (π) for *Haemoproteus* varied between 0.003 and 0.0038 (Table 1). *Plasmodium* showed a greater haplotype diversity in Colombia (1), followed by Argentina (0.86), Brazil (0.83), Chile (0.74) and Peru (0.69), with nucleotide diversity (π) varying between 0.003 and 0.049 (Table 1).

**Fig. 3:**
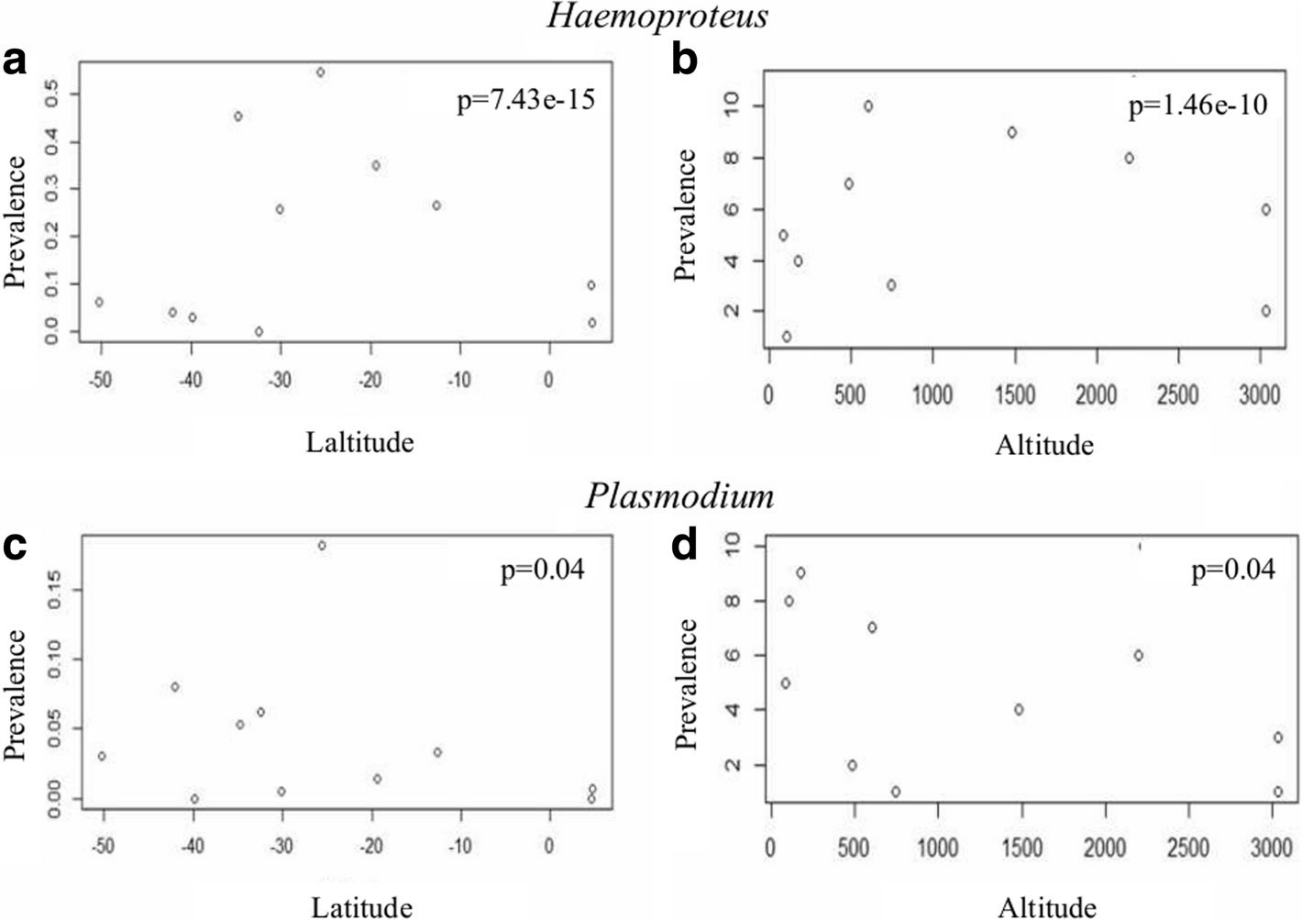
Dispersion diagram for *Haemoproteus* and *Plasmodium*. Dispersion diagram of relationship between *Haemoproteus* prevalence with latitude and altitude (**a** and **b**), and relationship between *Plasmodium* prevalence with latitude and altitude (**c** and **d**) in South America

The mean prevalence with confidence intervals grouped by country and geographical area used in our GLM analyses are shown in Additional file 3: Table S3. Results of our GLM analyses indicated that latitude and altitude had a significant effect on *Haemoproteus* (*P* < 0.001) and *Plasmodium* (*P* < 0.05) prevalence in South America (Table 2). The highest prevalence of *Haemoproteus* (Fig. 3a) and *Plasmodium* (Fig. 3c) was observed between 20 and 35°S (central Chile) and both genera decreased toward lower and higher latitude. *Haemoproteus* prevalence increased at higher altitudes up to approximately 2200 masl, where it began to decrease again (Fig. 3b) and *Plasmodium* prevalence increased at lower altitudes (Fig. 3d). Diversity was related neither to altitude nor latitude for either genus (Table 2).

**Table 2:** GLM analyses results *Haemoproteus* spp. and *Plasmodium* spp.

Response variable	GLM	Explanatory Variables	Coefficient	SE	*z*-value	*P*
*Haemoproteus*						
Total prevalence	Binomial	Altitude	-4.207e-04	6.564e-05	-6.409	1.46e-10***
		Latitude	0.15835	0.02036	-7.777	7.43e-15***
Total diversity	Poisson	Altitude	0.00006	0.00032	0.189	0.850
		Latitude	0.00793	0.02164	0.367	0.714
*Plasmodium*						
Total prevalence	Binomial	Altitude	-0.0003592	0.0001750	-2.053	0.0401*
		Latitude	-0.10548	0.05198	-2.029	0.0424*
Total diversity	Poisson	Altitude	0.00029	0.00961	0.031	0.983
		Latitude	-0.01224	0.56370	-0.022	0.975

*P < 0.05; ***P < 0.0001

*Abbreviations*: GLM, generalized linear model; SE, standard error

### Phylogenetic analysis

**Fig. 4:**
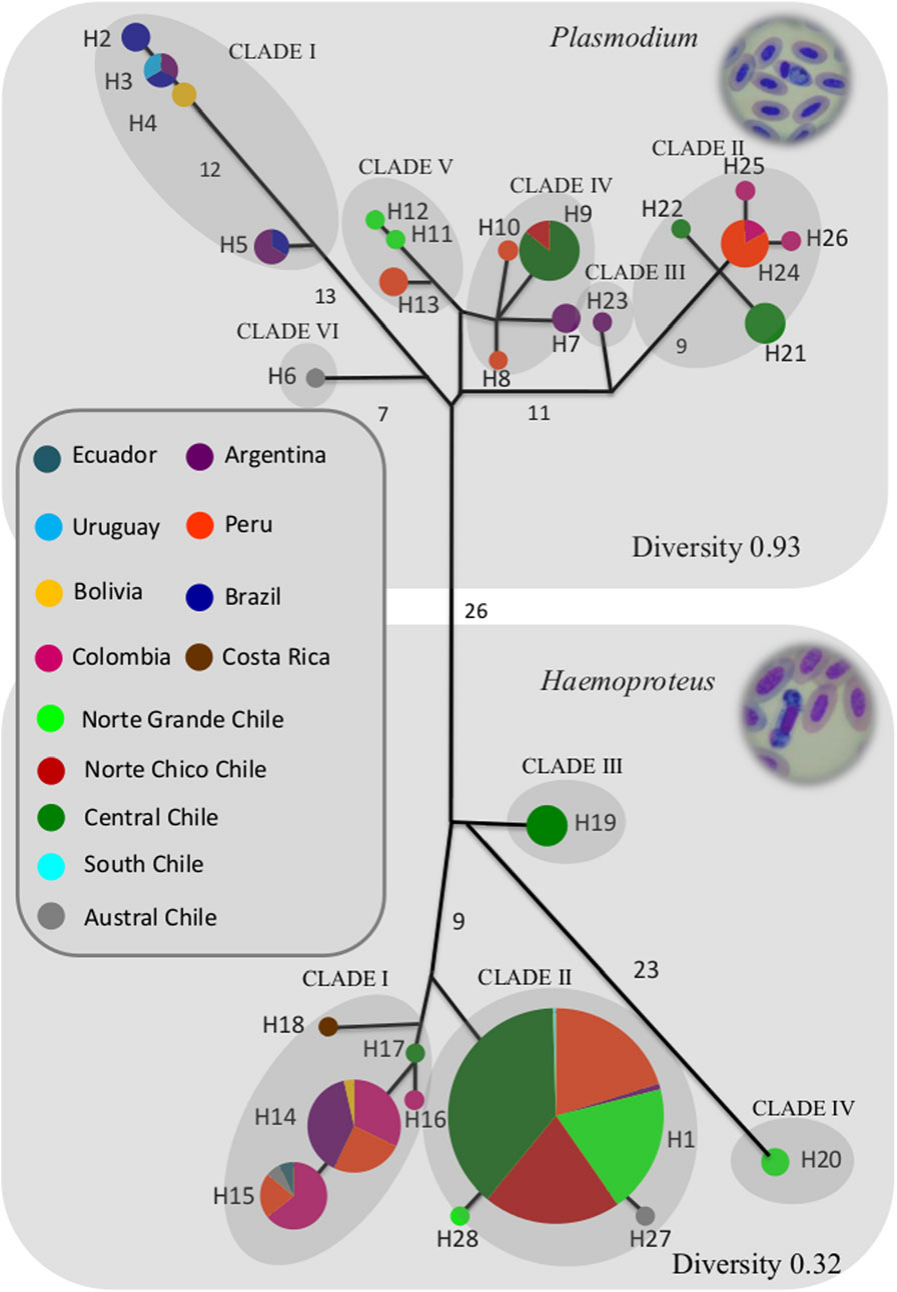
Median-joining network for *Haemoproteus* and *Plasmodium* from cytochrome *b* mtDNA. Each circle in the network corresponds to a different haplotype, the size of the circles correspond to haplotype frequencies, the numbers associated to each circle correspond to the number of haplotypes, and the colors of the circles correspond to the different countries

The Bayesian phylogenies for *cytb* of *Plasmodium* and *Haemoproteus* of Chile and South America showed similar patterns to those evident in the median-joining networks (MJN). Our phylogenetic analysis provided strong support for four clades in *Haemoproteus* and for six clades in *Plasmodium*. For *Haemoproteus*, Clade II includes haplotype 1, the most common in our survey. *Haemoproteus* shows distinct phylogeographical patterns, with Clade I generally located at lower latitudes, and with haplotype 14 showing some restriction in distribution caused by the Andes. For *Plasmodium*, Clade I clearly encompasses countries that are on the east side of the Andes (Brazil, Bolivia, Argentina and Uruguay). These countries correspond to temperate latitudinal zones with some sampling locations in the tropical zone (Brazil) and have warm temperatures (Fig. 4 and Additional file 4: Figure S1) [44].

### Parasite phylogeographical pattern

**Fig. 5:**
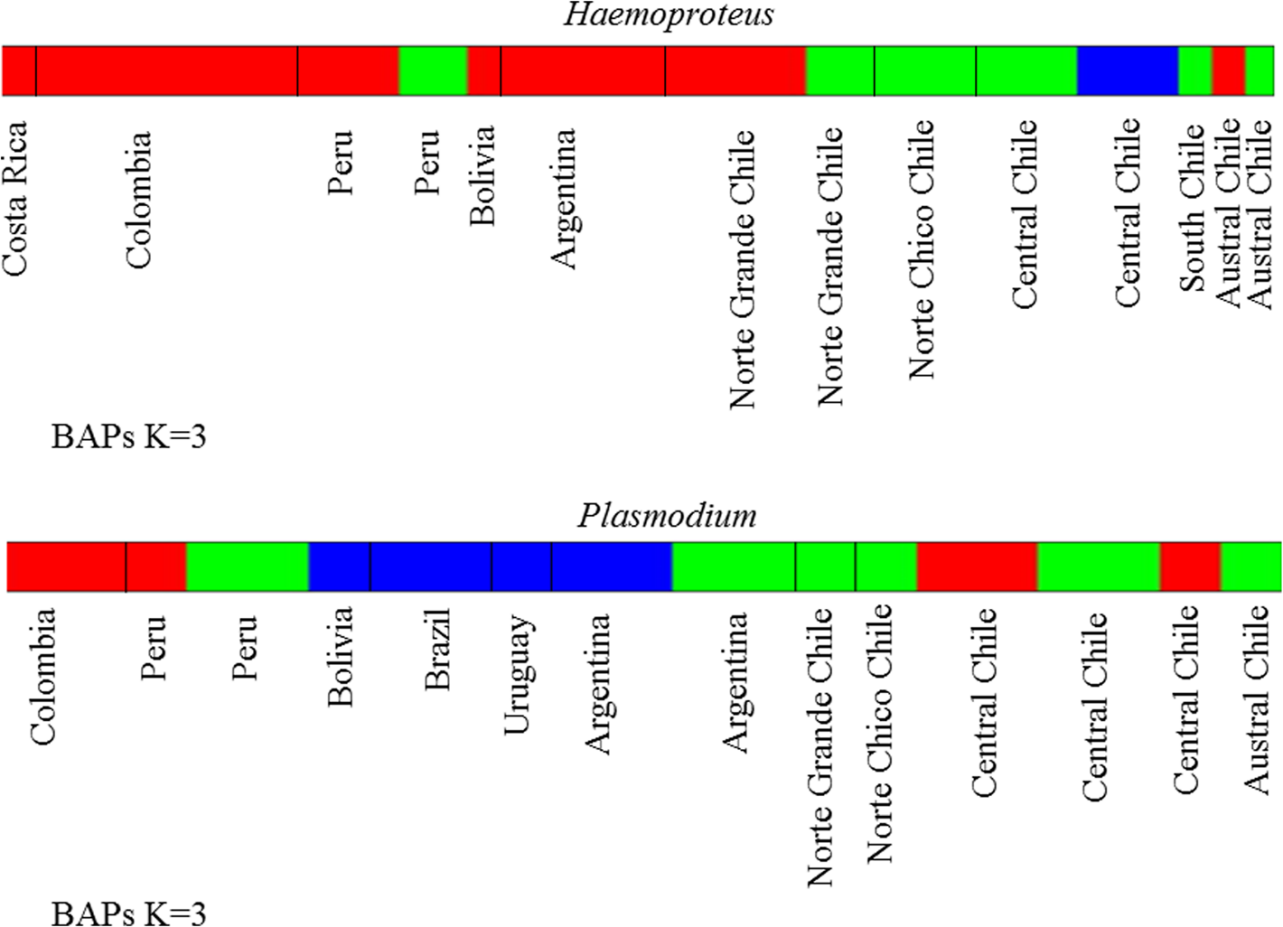
Bayesian analysis of population structure (BAPS). Type model population mixture analysis (spatial clustering of groups) shows 3 clusters (K = 3) for *Haemoproteus* and 3 clusters (K = 3) for *Plasmodium* spp.

Results of our BAPS analysis suggested three clusters (K = 3) for each genus (*Haemoproteus* and *Plasmodium*). For *Haemoproteus* the clusters corresponded to: (i) a region spanning Costa Rica to Norte Grande of Chile, including Bolivia, Argentina and Punta Arenas; (ii) an area encompassing the south of Peru, and locations in Chile such as south of Norte Grande, Chile, Norte Chico of Chile, and part of the Central area, Isla Mocha and Navarino islands; and (iii) a region that included central Chile (Termas del Flaco, Pantanillos and Parque Ingles). For *Plasmodium*, the clusters corresponded to: (i) Colombia, part of Peru and central Chile; (ii) part of Peru, Argentina and Chile; and (iii) Bolivia, Brazil, Uruguay and Argentina (Fig. 5).

**Fig. 6:**
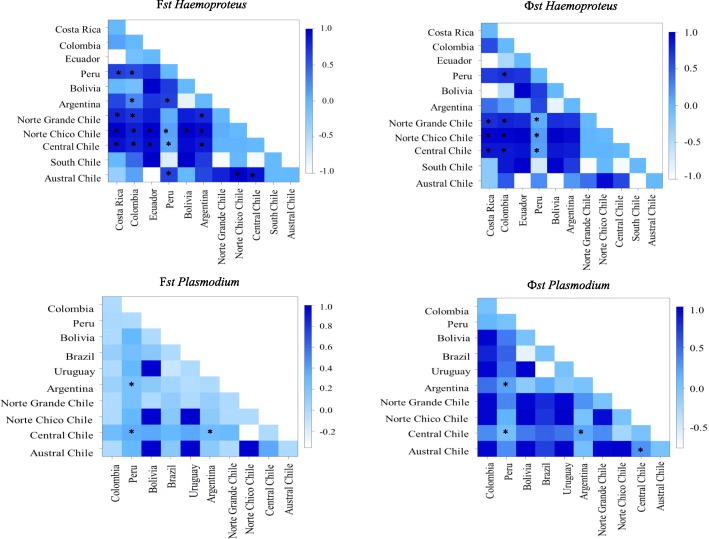
Pairwise Fst and Φ_ST_ values for cytochrome *b* DNA sequences. Fst and Φst values for countries and geographical zones of Chile. *P < 0.05

Of 55 pairwise F_ST_ values between mtDNA *Haemoproteus* from different locations, 21 were significantly different from zero, as were 15 of 55 Φ_ST_ values (*P* < 0.05) (Additional file 5: Table S4 and Additional file 6: Tables S5). Most of these were comparisons between sites in north and central Chile, and other locations (Fig. 6). For *Plasmodium*, only 3 of 45 comparisons were significantly different from zero for F_ST_, and 4 of 45 for Φ_ST_ (*P* < 0.05) (Additional file 7: Table S6 and Additional file 8: Table S7).

## Discussion

### Patterns of prevalence and geographical distribution

The overall prevalence of *Haemoproteus* and *Plasmodium* in rufous-collared sparrows across Central and South America was 25%, varying among localities from 0 to 100%. Differences in prevalence among sampling places may be attributable to several factors involved in the transmission of hemoparasites, including identity and diversity of vector and host species, and abiotic environmental factors like precipitation, mean annual temperature and seasonality [2].

The high overall prevalence was underlain principally by the presence of the most common haplotype of *Haemoproteus* (H1). This haplotype had a higher prevalence at locations between 32–33°S, similar to the findings of Merino et al. [23], who reported the highest prevalence between 33–35°S (locales Rinconada and Pantanillos, respectively). The prevalence of *Haemoproteus* and *Plasmodium* were significantly affected by latitude, where the highest prevalence was observed in the central region of Chile west of the Andes (20–25°S) decreasing toward lower and higher latitudes. East of the Andes, northern Argentina also showed high prevalence for both parasites. A lower prevalence at more southerly latitudes (34–42°S) may be the result of lower annual temperatures that can result in lower developmental rates of both vector [45] and parasite [1]. Furthermore, for *Haemoproteus* and *Plasmodium*, the significant effect of latitude along the western Andes of South America is primarily attributable to low prevalence in Colombia. This low prevalence and high diversity of *Haemoproteus* in Colombia (see González et al. [9]) could be explained by the variability of habitats and hosts. Regions with high potential host diversity, such as Colombia, can reduce disease risk since pathogens are apportioned among many different hosts [46]. This may explain the potential dilution effect [47] for low parasite prevalence for rufous-collared sparrows found in Colombia.

Elevation has been suggested as a limiting factor for *Plasmodium* distribution due to lower temperatures at higher altitudes [10, 48, 49], resulting in a diminution of vectors with increasing elevation [10]. Imura et al. [3] attributed the low prevalence of *Plasmodium* and *Haemoproteus* among wild birds to the diminished abundance or even absence of appropriate vectors at high altitudes. In our study, we failed to detect *Plasmodium* above 600 m of altitude in Chile, Bolivia, Argentina, Brazil and Uruguay, consistent with this assertion. *Plasmodium* appears to be more sensitive to lower temperatures, with an optimal range of diurnal temperatures from 18 to 24 °C for development within vectors [50]. However, *Haemoproteus* prevalence increases with altitude, similar to patterns detected by Rooyen et al. [10], and declines above approximately 2000 m above sea level. Olsson-Pons et al. [51] suggested that infection patterns for hemoparasites are best predicted by geographical and abiotic factors for *Plasmodium*, but that host-parasite interactions are more important for predicting *Haemoproteus*.

### Parasite diversity and distribution

Our study indicated a high genetic diversity for *Plasmodium*, but low genetic diversity for *Haemoproteus* in rufous-collared sparrows. Although diversity estimates were not statistically significantly related to altitude or latitude, a higher clade diversity (or lineages) was observed for both genera at lower latitudes.

This latitudinal diversity gradient may relate to temperature and precipitation, as these are abiotic variables that are known to enhance parasite diversification [52], but also to predict diversity of parasite hosts (birds and vectors). However, a recent study reported no influence of latitude or climate variation on the phylogenetic diversity of *Haemoproteus* and *Plasmodium* [16].

The proportionately higher diversity of *Plasmodium* compared to *Haemoproteus* (see also [15]), has been previously documented in rufous-collared sparrows [9, 24–28]. This difference in diversity may be caused by a lower specificity of *Plasmodium* for their host, but also because *Plasmodium* diversification is more likely influenced by host-switching [53]. Such host-switching would not produce a stable relationship over time [33], and thus would preclude the evolution of specialization. Thus, we can infer that the higher haplotype diversity in some sampled regions may relate to a greater number of potential avian host species.

Several lineages of *Haemoproteus* and *Plasmodium* that we found in rufous-collared sparrows have been reported by other authors [9, 23–28]. Moreover, some of these parasite lineages have been found in other passerine species, which suggests some host-switching [17, 33] and a lack of host species specificity. Lineages of both parasite genera contain examples of specialization and generalism [33, 54]; however, multiple studies indicate that *Haemoproteus* is typically more host-specific than *Plasmodium* [4, 33, 55] and generally more constrained at the host family level [33]. For instance, Merino et al. [23] suggested that *Haemoproteus* is typically found within the passerine family Emberizidae, the family to which the rufous-collared sparrow belongs.

*Haemoproteus* haplotype H1 was the dominant haplotype in populations from Chile and Peru (see also [24]). Such a high prevalence and wide geographical distribution of a parasite implies parasite-host co-adaptation. This observation may also imply that haplotype H1 is endemic to those portions of South America. Endemic avian haemosporidian species tend to cause chronic disease with low virulence [1]. However the previously-noted difference between *Haemoproteus* and *Plasmodium* could be shifting as *Haemoproteus* shows signs of evolution from specialist to generalist tendencies in South America [56]; this might help explain the elevated genetic diversity of *Haemoproteus* that we found.

### Parasite phylogeographical pattern

We found greater diversity in *Plasmodium* than *Haemoproteus* across surveyed regions, with a tendency to greater diversity at lower latitudes for both genera. For *Haemoproteus* we found a single dominant haplotype, but in both taxa we documented geographical patterns in the distribution of parasite lineages. For *Haemoproteus* we found a clear phylogeographical boundary in Peru. Interestingly, a similar phylogeographical boundary has been described for the avian host, with different rufous-collared sparrow haplogroups in Peru and Chile [22]. Aside from this boundary, the distributions of *Haemoproteus* and *Plasmodium* haplogroups in Central and South America do not seem to show patterns that are coincident with those present in the rufous-collared sparrow [22, 57]. Co-divergence histories of haemosporidian parasites with their avian hosts is dominated by host-switching events, and co-speciation is mostly observed at the family level rather than at the host population or species level [58].

One *Haemoproteus* haplotype (H15) was found in the austral region in Chile, and in northern countries (Peru, Ecuador, Colombia and Costa Rica), but was absent in the remaining sampled areas. This odd disjunction might be caused by avian migration, especially as the southernmost portion of Chile that corresponds to an overlap between two main migratory routes between the Northern and Southern Hemispheres [59]. Furthermore, bird migration has contributed to the wide distribution of haemosporidian parasites [1]. A major biogeographical boundary for avian species in South America is the Andes Mountains [60, 61]. Although results from our BAPs analysis (Fig. 5) grouped the samples from Argentina with the northern clade, there is a clear distinction in haplotype distribution between regions with a higher frequency of the haplotype H14. For *Plasmodium*, a distinct clade, consistent with results from BAPs that show Argentina, Uruguay, Brazil and Bolivia (Fig. 5) grouped together, supports the notion that the Andean massif limits gene flow in these parasites. Such assertions are preliminary and sampling of a greater geographical intensity is required for the eastern part of the Andes. Limited genealogical structure in *Plasmodium* across the remaining studied locations might be associated with a tendency towards host-parasite generalists with marked gene flow among different hosts, but this might also be a consequence of relatively low sample sizes, again meriting further study with larger arrays of samples.

## Conclusions

The prevalence of *Haemoproteus* was markedly higher than *Plasmodium*, in contrast to patterns of haplotype diversity. This dichotomous observation may be attributable to the greater host specificity of *Haemoproteus* relative to *Plasmodium*. In South America, *Haemoproteus* and *Plasmodium* showed latitudinal and altitudinal patterns, with a prevalence peak between 20–40°S, followed by a decrease at higher latitudes. We found that *Plasmodium* prevalence increased at lower altitudes while *Haemoproteus* prevalence increased at higher altitudes. Our study is the first of *Plasmodium* and *Haemoproteus* for many of these regions in Latin America, and provides a map of hemoparasite prevalence and diversity within one of the most broadly-distributed passerine species in the world. Future studies should examine the prevalence of hemoparasites in other species of passerines, providing further information on parasite-host specificity. Our study adds to the current knowledge of prevalence and diversity of haemosporidian parasites. Low temperatures of the higher elevations can contribute to reduce the presence of avian hemosporidia and vectors. An increase in temperature due to climatic change could result in an increase in the latitudinal and altitudinal ranges of *Haemoproteus* and *Plasmodium*. This knowledge will also be useful in disease risk assessment for avian populations for their conservation.

## Additional files

Additional file 1: **Table S1.** Avian haemosporidian haplotypes prevalence with country, locality, latitude, longitude and altitude. (DOCX 45 kb)

Additional file 2: **Table S2.** Avian haemosporidian haplotypes used in phylogenetic reconstruction, with GenBank accession number and country. (DOCX 49 kb)

Additional file 3: **Table S3.** Prevalence and confidence intervals by country and geographical area grouped for the GLM analysis. (DOCX 78 kb)

Additional file 4: **Figure S1.** Bayesian phylogenetic reconstructions of *Haemoproteus* and *Plasmodium* species with available *cyt b* sequences (441 bp). Posterior probabilities of branch support are shown. Outgroup taxa correspond to *Leucocytozoon toddy. (DOCX 224 kb)*

Additional file 5: **Table S4.** Pairwise F_st_ values calculated from mtDNA *Haemoproteus* sequences between countries and geographical areas of Chile. (DOCX 45 kb)

Additional file 6: **Table S5.** Pairwise Φst values calculated from mtDNA *Haemoproteus* sequences between countries and geographical areas of Chile. (DOCX 44 kb)

Additional file 7: **Table S6.** Pairwise Fst values calculated from mtDNA *Plasmodium* sequences between countries and geographical areas of Chile. (DOCX 43 kb)

Additional file 8: **Table S7.** Pairwise Φst values calculated from mtDNA *Plasmodium* sequences between countries and geographical areas of Chile. (DOCX 43 kb)

## Abbreviations

CI: Confidence interval
GD: Decimal degrees
GLMs: Generalized lineal models
H: Haplotype
Haem: *Haemoproteus*
Hd: Gene diversity
masl: Meters above sea level
Max: Maximum
Min: Minimum
N: Sample size
nH: Haplotype number
Plas: *Plasmodium*
S: Number of polymorphic sites
π: Nucleotide diversity
