# Supplementary material for: Spatial distribution, prevalence and diversity of haemosporidians in the rufous-collared sparrow, Zonotrichia capensis
Source: Parasit Vectors. 2019 Jan 3;12:2. doi: 10.1186/s13071-018-3243-4 (PMC6318949; doi:10.1186/s13071-018-3243-4)
Supplement: Supplementary file 1 — Table S1. Avian haemosporidian haplotypes prevalence with country, locality, latitude, longitude and altitude. (DOCX 45 kb) [file 13071_2018_3243_MOESM1_ESM.docx]

**Additional file 1:** **Table S1.** Avian haemosporidian haplotypes prevalence with country, locality, latitude, longitude and altitude

| Country | N N | Locality | Latitude | Longuitude | Altitude | n | n total | n Haem | Prevalence | n Plas | Prevalence | Prevalence | Haplotype name |
| --- | --- | --- | --- | --- | --- | --- | --- | --- | --- | --- | --- | --- | --- |
|  | map |  | (GD) | (GD) | m asl | Total | Positive | Positive | (%) Haem | Positive | (%) Plas | % total | (n positive samples) |
| Costa Rica | 1 1 | La Georgina, San José | 9.56 | -83.73 | 2996 | 2 | 2 | 2 | 100 | 0 | 0 | 100 | H18/H15 |
| Colombia | 2 2 | Pereira | 4.7 | -75.48 | 1290 | - | 1 | 0 | 0 | 1 | 0 | 0 | H24 |
|  | 3 2 | Los Nevavos NNP, Otún Lagoon | 4.76 | -75.4 | 3950 | 22 | 0 | 0 | 0 | 0 | 0 | 0 | No haplotype |
|  | 4 2 | Los Nevavos NNP, El bosque station | 4.76 | -75.45 | 3300 | 66 | 0 | 0 | 0 | 0 | 0 | 0 | No haplotype |
|  | 5 2 | Ucumari RNP, La Pastora | 4.71 | -75.48 | 2400 | 72 | 4 | 2 | 2.8 | 2 | 2.8 | 5.6 | H25(1)/H26(1)H14(2) |
|  | 6 2 | Grasslands and High Andean forest (E) | 4.71 | -75.45 | 2900 | 44 | 0 | 0 | 0 | 0 | 0 | 0 | No haplotype |
|  | 7 2 | Paramo (E) | 4.76 | -75.4 | 4000 | 14 | 0 | 0 | 0 | 0 | 0 | 0 | No haplotype |
|  | 8 3 | Ucumari RNP, El Cedral | 4.71 | -75.56 | 2100 | 33 | 1 | 1 | 3 | 0 | 0 | 3 | H14 |
|  | 9 3 | Wildlife Sanctuary Otún Quimbaya | 4.71 | -75.56 | 1850 | 28 | 1 | 1 | 3.6 | 0 | 0 | 3.6 | H14 |
|  | 10 4 | Gimnasio Colombo Britanico School | 4.8 | -74.06 | 2560 | 2 | 1 | 1 | 50 | 0 | 0 | 50 | H15 |
|  | 11 5 | UN de Colombia-Bogotá | 4.63 | -74.08 | 2560 | 76 | 8 | 8 | 10.5 | 0 | 0 | 10.5 | (5)H15/(3)H14 |
|  | 12 5 | Cundinamarca- Bogotá | 4.63 | -74.08 | 2650 | 36 | 5 | 5 | 13.9 | 0 | 0 | 13.9 | (2)H14/(1)H16/(2)H15 |
|  | 13 6 | Chingaza NNP, Palacio Forest | 4.68 | -73.83 | 2900 | 18 | 1 | 1 | 5.6 | 0 | 0 | 5.6 | H15 |
|  | 14 6 | Chingaza NNP, Monterredondo | 4.61 | -73.71 | 3100 | 8 | 0 | 0 | 0 | 0 | 0 | 0 | No haplotype |
|  | 15 6 | Paramo (E) | 4.61 | -73.71 | 3100 | 8 | 0 | 0 | 0 | 0 | 0 | 0 | No haplotype |
| Ecuador | 16 7 | Podocarpus NP, Cerro Toledo | -4.6 | -79.23 | 2500 | 1 | 1 | 1 | 100 | 0 | 0 | 100 | H15 |
| Peru | 17 8 | Huanuco | -9.89 | -76.54 | 3015 | 3 | 1 | 1 | 33.3 | 0 | 0 | 33.3 | H14 |
|  | 18 9 | Huanuco | -9.91 | -76.23 | 3077 | 23 | 14 | 7 | 30.4 | 7 | 30.4 | 60.9 | (5)H24/(6)H14/H13(2)/(1)H15 |
|  | 19 10 | Oxampa | -10.56 | -75.4 | 2953 | 1 | 1 | 1 | 100 | 0 | 0 | 100 | H15 |
|  | 20 11 | Huancavelica | -12.76 | -74.98 | 4655 | 184’ | 1 | 1 | - | 0 | - | - | H1 |
|  | 21 12 | Ica | -13.98 | -75.5 | 449 | 184’ | 46 | 46 | - | 0 | - | - | (46)H1 |
|  | 22 13 | Ica | -14.13 | -76.35 | 672 | 184’ | 1 | 0 | - | 1 | - | - | H8 |
|  | 23 14 | Tacna | -17.6 | -70.23 | 590 | 184’ | 1 | 0 | - | 1 | - | - | H10 |
| Bolivia | 24 15 | La Paz | -16.3 | -67.78 | 3067 | 1 | 1 | 1 | 100 | 0 | 0 | 100 | H14 |
|  | 25 16 | Catarata Arcoiris, Sta. Cruz | -13.91 | -60.75 | 467 | 2 | 1 | 0 | 0 | 1 | 50 | 50 | H4 |
|  | 26 17 | Charagua, Sta Cruz | -19.80 | -61.84 | 382 | 3 | 0 | 0 | 0 | 0 | 0 | 0 | No haplotype |
| Brazil | 27 18 | Serido | -6.88 | -36.45 | 594 | - | 2 | 0 | 0 | 2 | 0 | 0 | H2(2) |
|  | 28 19 | Aracruz | -19.81 | -40.26 | 14 | - | 2 | 0 | 0 | 2 | 0 | 0 | H3/H5 |
| Uruguay | 29 20 | Uruguay | -32.45 | -55.75 | 104 | 16 | 1 | 0 | 0 | 1 | 6.25 | 6.25 | H3 |
| Argentina | 30 21 | Yavi, Jujuy | -22.13 | -65.46 | 3848 | 6 | 6 | 6 | 100 | 0 | 0 | 100 | H14(6) |
|  | 31 22 | Tres cruces, Jujuy | -22.91 | -65.58 | 3721 | 3 | 2 | 2 | 66.7 | 0 | 0 | 66.7 | H14 |
|  | 32 23 | El Peñón, Catamarca | -26.07 | -67.18 | 3779 | 1 | 0 | 0 | 0 | 0 | 0 | 0 | No haplotype |
|  | 33 24 | Tafí del Valle, Tucumán | -26.86 | -67.18 | 3817 | 4 | 3 | 3 | 75 | 0 | 0 | 75 | H14(3) |
|  | 34 25 | Quimilí, Santiago del Estero | -27.63 | -62.59 | 128 | 5 | 3 | 1 | 20 | 2 | 40 | 60 | H5(2)H1 |
|  | 35 26 | RN del Iberá, Corrientes | -28.1 | -57.1 | 67 | 2 | 1 | 0 | 0 | 1 | 50 | 50 | H3 |
|  | 36 27 | Iguazú, Misiones | -25.56 | -54.56 | 229 | 1 | 1 | 0 | 0 | 1 | 100 | 100 | H7 |
|  | 37 28 | Mendoza | -32.87 | -68.82 | 743 | 1 | 0 | 0 | 0 | 0 | 0 | 0 | No haplotype |
|  | 38 29 | La Reja, Buenos Aires | -34.67 | -58.83 | 27 | 1 | 0 | 0 | 0 | 0 | 0 | 0 | No haplotype |
|  | 39 30 | Magdalena, Buenos Aires | -35.07 | -57.54 | 21 | 5 | 2 | 0 | 0 | 2 | 40 | 40 | H7(1)H23(1) |
|  | 40 31 | Trelew, Chunut | -43.25 | -65.3 | 207 | 5 | 1 | 1 | 20 | 0 | 0 | 20 | H1 |
|  | 41 32 | Güer Aike, Santa Cruz | -51.66 | -69.58 | 48 | 7 | 0 | 0 | 0 | 0 | 0 | 0 | No haplotype |
|  | 42 33 | Ushuaia, Tierra del Fuego | -54.81 | -68.30 | 27 | 6 | 0 | 0 | 0 | 0 | 0 | 0 | No haplotype |
| Chile | 43 34 | Caleta Vitor | -18.75 | -70.33 | 30 | 12 | 3 | 1 | 8.3 | 2 | 16.7 | 25 | H1/H11/H12 |
| Norte Grande | 44 35 | Valle Lluta | -18.40 | -69.99 | 812 | 15 | 0 | 0 | 0 | 0 | 0 | 0 | No haplotype |
|  | 45 36 | Azapa | -18.50 | -70.24 | 156 | 23 | 3 | 3 | 13 | 0 | 0 | 13 | H1(3) |
|  | 46 37 | Putre | -18.19 | -69.55 | 1666 | 12 | 7 | 7 | 58.3 | 0 | 0 | 58.3 | H1 |
|  | 47 38 | Socoroma | -18.26 | -69.60 | 1643 | 53 | 35 | 35 | 66 | 0 | 0 | 66 | (31)H1/(1)H17/(2)H20/(1)H28 |
|  | 48 39 | Chusmiza | -19.68 | -69.19 | 3468 | 4 | 0 | 0 | 0 | 0 | 0 | 0 | No haplotype |
|  | 49 40 | Pampa del Tamarugal | -20.47 | -69.67 | 990 | 19 | 2 | 2 | 10.5 | 0 | 0 | 10.5 | H1(2) |
|  | 50 41 | Quebrada del Jere | -23.18 | -67.99 | 3119 | 2 | 1 | 1 | 50 | 0 | 0 | 50 | H1 |
| Chile | 51 42 | PN Llanos de Challe | -28.14 | -71.12 | 400 | 20 | 8 | 8 | 40 | 0 | 0 | 40 | H1(8) |
| Norte Chico | 52 43 | PN Fray Jorge | -30.65 | -71.69 | 34 | 134 | 29 | 28 | 20.9 | 1 | 0.7 | 21.6 | H1(28)/H9(1) |
|  | 53 44 | RN Las Chinchillas | -31.55 | -71.10 | 1018 | 33 | 12 | 12 | 36.4 | 0 | 0 | 36.4 | H1(12) |
| Chile | 54 45 | PN La Campana | -32.96 | -71.08 | 851 | 33 | 22 | 22 | 66.7 | 0 | 0 | 66.7 | H1(22) |
| Central | 55 45 | Cerro el Roble | -32.97 | -71.01 | 725 | 1 | 0 | 0 | 0 | 0 | 0 | 0 | No haplotype |
|  | 56 46 | Til-Til | -33.08 | -70.92 | 588 | 68 | 61 | 60 | 88.2 | 1 | 1.5 | 89.7 | H1(60)/H22(1) |
|  | 57 47 | Lago Peñuelas | -33.16 | -71.46 | 348 | 9 | 1 | 0 | 0 | 1 | 11.1 | 11.1 | H21 |
|  | 58 48 | Rinconada | -33.53 | -70.1 | 567 | 37 | 14 | 8 | 21.6 | 6 | 16.2 | 37.8 | (8)H1/(6)H9 |
|  | 59 49 | RN El Yali | -33.75 | -71.70 | 21 | 6 | 0 | 0 | 0 | 0 | 0 | 0 | No haplotype |
|  | 60 50 | Termas del Flaco | -34.96 | -70.43 | 1735 | 16 | 1 | 1 | 6.3 | 0 | 0 | 6.3 | H19 |
|  | 61 51 | Sierra de Bellavista | -34.80 | -70.75 | 1033 | 4 | 0 | 0 | 0 | 0 | 0 | 0 | No haplotype |
|  | 62 52 | Pantanillos | -35.41 | -72.13 | 132 | 8 | 4 | 2 | 25 | 2 | 25 | 50 | (2)H19/H21(2) |
|  | 63 53 | La Mina | -35.98 | -70.40 | 1105 | 5 | 0 | 0 | 0 | 0 | 0 | 0 | No haplotype |
|  | 64 54 | Parque Inglés | -35.28 | -71.14 | 1097 | 5 | 1 | 1 | 20 | 0 | 0 | 20 | H19 |
|  | 65 55 | Reloca | -36.39 | -72.47 | 80 | 5 | 0 | 0 | 0 | 0 | 0 | 0 | No haplotype |
|  | 66 56 | Laguna Santa Elena | -36.80 | -72.38 | 75 | 9 | 1 | 0 | 0 | 1 | 11.1 | 11.1 | H21 |
|  | 67 57 | Shangrilá | -36.91 | -71.48 | 154 | 1 | 0 | 0 | 0 | 0 | 0 | 0 | No haplotype |
| Chile | 68 58 | PN Nahuelbuta | -37.79 | -72.99 | 1300 | 1 | 0 | 0 | 0 | 0 | 0 | 0 | No haplotype |
| South | 69 59 | Isla Mocha | -38.35 | -73.92 | 320 | 26 | 1 | 1 | 3.8 | 0 | 0 | 3.8 | H1 |
|  | 70 60 | Maicolpué | -40.55 | -73.17 | 71 | 3 | 0 | 0 | 0 | 0 | 0 | 0 | No haplotype |
|  | 71 61 | PN Puyehue | -40.69 | -72.11 | 609 | 1 | 0 | 0 | 0 | 0 | 0 | 0 | No haplotype |
|  | 72 62 | Hornopirén | -41.91 | -72.26 | 1424 | 4 | 0 | 0 | 0 | 0 | 0 | 0 | No haplotype |
| Chile | 73 63 | PN Chiloé | -42.625 | -74.10 | 10 | 7 | 1 | 0 | 0 | 1 | 14.3 | 14.3 | H6 |
| Austral | 74 64 | Punta Arenas | -53.13 | -70.91 | 28 | 6 | 1 | 1 | 16.7 | 0 | 0 | 16.7 | H15 |
|  | 75 65 | Navarino | -54.93 | -67.6 | 215 | 20 | 1 | 1 | 5 | 0 | 0 | 5 | H27 |

Prevalence of avian haemosporidian parasite genera (*Plasmodium* & *Haemoproteus*) in rufous collared sparrows (*Zonotrichia capensis*) separated by country and geographical área. The “%” sign represents prevalence of birds infected with avian haemosporidian and “*n*” indicates the number of individuals.

*Abbreviations:* N: number of locations; N map: number of locations on the map; NNP: National Natural Park. RNP: Regional Natural Park: (E); Ecocystems PN: National Park; RN: National Reserve; UN: National University; GD: decimal degrees; m asl: meters above sea level; H: haplotype; Haem: *Haemoproteus*; Plas: *Plasmodium;* 184’ [24]; total study number; n: number of samples.
