# Supplementary material for: Spatial distribution, prevalence and diversity of haemosporidians in the rufous-collared sparrow, Zonotrichia capensis
Source: Parasit Vectors. 2019 Jan 3;12:2. doi: 10.1186/s13071-018-3243-4 (PMC6318949; doi:10.1186/s13071-018-3243-4)
Supplement: Supplementary file 2 — Table S2. Avian haemosporidian haplotypes used in phylogenetic reconstruction, with GenBank accession number and country. (DOCX 49 kb) [file 13071_2018_3243_MOESM2_ESM.docx]

**Additional file 2:** **Table S2** Avian haemosporidian haplotypes prevalence with country, locality, latitude, longitude and altitude.

*Haplotype detected in this study.

*Abbreviations:* H, haplotype
