# Supplementary material for: Spatial distribution, prevalence and diversity of haemosporidians in the rufous-collared sparrow, Zonotrichia capensis
Source: Parasit Vectors. 2019 Jan 3;12:2. doi: 10.1186/s13071-018-3243-4 (PMC6318949; doi:10.1186/s13071-018-3243-4)
Supplement: Supplementary file 3 — Table S3. Prevalence and confidence intervals by country and geographical area grouped for the GLM analysis. (DOCX 78 kb) [file 13071_2018_3243_MOESM3_ESM.docx]

**Additional file 3: Table S3** Prevalence and confidence intervals by country and geographical area grouped for the GLM analysis.

*Abbreviations:* N map: number of locations on the map; Haem: *Haemoproteus*; Plas: *Plasmodium;* n: number of samples; Min:minimum; Max: maximum; CI: confidence interval.
