# Supplementary material for: Spatial distribution, prevalence and diversity of haemosporidians in the rufous-collared sparrow, Zonotrichia capensis
Source: Parasit Vectors. 2019 Jan 3;12:2. doi: 10.1186/s13071-018-3243-4 (PMC6318949; doi:10.1186/s13071-018-3243-4)

**Additional file 4: Figure S1 Bayesian phylogenetic reconstructions of *Haemoproteus* and *Plasmodium.*** Bayesian phylogenetic reconstructions of *Haemoproteus* and *Plasmodium* species with available *cyt b* sequences (441bp). Posterior probabilities of branch support are shown. Outgroup taxa correspond to *Leucocytozoon toddy*


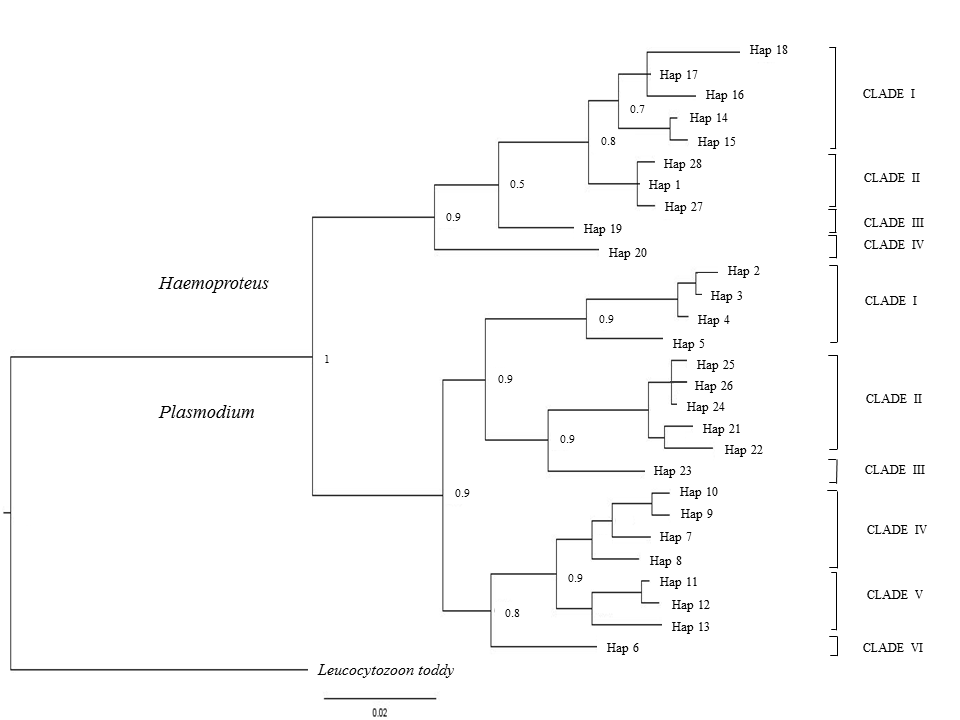

Supplement: Supplementary file 4 — Figure S1. Bayesian phylogenetic reconstructions of Haemoproteus and Plasmodium species with available cyt b sequences (441 bp). Posterior probabilities of branch support are shown. Outgroup taxa correspond to Leucocytozoon toddy. (DOCX 224 kb) [file 13071_2018_3243_MOESM4_ESM.docx]
