# Supplementary material for: Spatial distribution, prevalence and diversity of haemosporidians in the rufous-collared sparrow, Zonotrichia capensis
Source: Parasit Vectors. 2019 Jan 3;12:2. doi: 10.1186/s13071-018-3243-4 (PMC6318949; doi:10.1186/s13071-018-3243-4)
Supplement: Supplementary file 8 — Table S7. Pairwise Φst values calculated from mtDNA Plasmodium sequences between countries and geographical areas of Chile. (DOCX 43 kb) [file 13071_2018_3243_MOESM8_ESM.docx]

**Additional file 8:** **Table S7** Pairwise Φst values calculated from mtDNA *Plasmodium* sequences between countries and geographic areas of Chile.

Significance of population differences based on a 1000 permutations: *p < 0.05
